# ASSESSING AGE-RELATED CHANGES IN SKELETAL MUSCLE PERFUSION AND MICROVASCULAR FUNCTION WITH MRI

**DOI:** 10.1093/geroni/igad104.1280

**Published:** 2023-12-21

**Authors:** David Reiter

**Affiliations:** Emory University, Atlanta, Georgia, United States

## Abstract

MRI affords a wide variety of non-invasive and quantitative approaches for characterizing tissue perfusion, each providing distinct strengths and weaknesses. Skeletal muscle exhibits a tremendous range of metabolic activity with up to a 50-fold increase in oxygen consumption between resting state and heavy exercise. Even at rest, perfusion of skeletal muscle is a dynamic process where blood flow delivery to capillary beds fluctuates and is regulated by a host of cellular, metabolic, and neurovascular inputs. This talk will present an overview of current and emerging MRI methodologies used for characterizing skeletal muscle perfusion and microvascular function. Strengths and weaknesses of each methodology will be presented with emphasis on studies in aging and age-related disease.

